# RETRACTION of: Overexpression of *VP*, a vacuolar H^+^-pyrophosphatase gene in wheat (*Triticum aestivum* L.), improves tobacco plant growth under Pi and N deprivation, high salinity, and drought

**DOI:** 10.1093/jxb/erw149

**Published:** 2016-05-07

**Authors:** Xiaojuan Li, Chengjin Guo, Juntao Gu, Weiwei Duan, Miao Zhao, Chunying Ma, Xiaoming Du, Wenjing Lu, Kai Xiao

**Affiliations:** ^1^College of Agronomy, Agricultural University of Hebei, Baoding 071001, China; ^2^College of Life Sciences, Agricultural University of Hebei, Baoding 071001, China; ^3^Science and Technology College, North China Electric Power University, Baoding 071051, China

*Journal of Experimental Botany* Vol 65 (2); 2014 pp. 683–696.

doi: 10.1093/jxb/ert442

The Editor-in-Chief and Publisher have retracted this article due to concerns relating to the validity of several figures:

- **Figure 2A** and **B**. The two bands used to represent *TaVP* expression in 0hr & 12hr high salinity and 0hr & 12hr drought treatments in root tissue appear to be duplicates, as are the three tubulin bands used as controls for root tissue and leaf tissue
- **Figure 3A**. *TaVP* expression. The same control band has been used nine different times to represent all eight lines and the WT control.
- Additionally both figures mentioned above have been individually constructed from different gels/blots. Journal policy requires this should have been made explicit in the construction of the figure (i.e. by using dividing lines or spaces) and in the figure legend.

The *Journal* applied forensic image analysis to verify the above irregularities, and also obtained advice from experts in the field. It is felt that the falsification of these images raises sufficient doubt over subsequent interpretations of data reported in the paper and as such the decision has been taken to retract.

